# Taxonomic Revision of the Genus *Miltotranes* Zimmerman, 1994 (Coleoptera: Curculionidae: Molytinae), the *Bowenia*-Pollinating Cycad Weevils in Australia, with Description of a New Species and Implications for the Systematics of *Bowenia* †

**DOI:** 10.3390/insects13050456

**Published:** 2022-05-12

**Authors:** Yun Hsiao, Rolf G. Oberprieler

**Affiliations:** 1 Division of Ecology and Evolution, Research School of Biology, The Australian National University, Canberra, ACT 2601, Australia; 2 Australian National Insect Collection, CSIRO, Canberra, ACT 2601, Australia; rolf.oberprieler@csiro.au or

**Keywords:** Australia, cycad systematics, cycad pollinators, new taxa, taxonomy, conservation

## Abstract

**Simple Summary:**

*Miltotranes* Zimmerman, 1994 is a genus of weevils pollinating *Bowenia* cycads belonging to two small endemic Australian species included in the IUCN Red List of Threatened Species and CITES Appendix II. We provide the first taxonomic revision of *Miltotranes* resulting in the identification of two previously described species and a newly recognised species, *M. wilsoni* sp. n. from the McIlwraith Range of the Cape York Peninsula. Morphological comparison reveals its affinity to *Tranes*, whose species are the pollinators of *Macrozamia* and *Lepidozamia* cycads. It appears that the association of *Miltotranes* with *Bowenia* may represent a secondary host switch in the *Tranes* group due to a closer relationship between *Macrozamia*, *Lepidozamia* and African *Encephalartos* than with *Bowenia*. The coincidence of the geographic ranges of *Miltotranes* weevils with distribution of their cycad hosts indicates that the isolated *Bowenia* population in the McIlwraith Range may represent a distinct, third species of *Bowenia*. Likely, the conspecificity of the *Miltotranes* weevils occurring in the Wet Tropics also suggests that several morphologically enigmatic localised populations represent *B. spectabilis*, confirming earlier botanical conclusions. The present study highlights the significance of systematic study of tightly plant-associated insects and its relevance for the taxonomy of their host plants.

**Abstract:**

The Australian endemic weevils of the genus *Miltotranes* Zimmerman, 1994 (Curculionidae: Molytinae: *Tranes* group), comprising two species, *M. prosternalis* (Lea, 1929) and *M. subopacus* (Lea, 1929), are highly host-specific and the only known pollinators of *Bowenia* cycads, which comprise two CITES-protected species restricted to Tropical Queensland in Australia. In the present study, the taxonomy of *Miltotranes* is reviewed, a lectotype for the name *Tranes prosternalis* Lea, 1929 is designated and a new species associated with the *Bowenia* population in the McIlwraith Range is described as *M. wilsoni* sp. n. The descriptions and diagnoses of all species are supplemented with illustrations of their habitus and salient structures, and an identification key to all species and a distribution map are provided. Potential implications of the new species and of the taxonomy and biogeography of *Miltotranes* overall on the systematics and conservation of *Bowenia* are discussed.

## 1. Introduction

*Miltotranes* Zimmerman, 1994 (Curculionidae: Molytinae: *Tranes* group) is a genus of weevil endemic to north-eastern continental Australia, currently comprising two named species, which have evolved an obligate pollination mutualism with *Bowenia* [1,2,3,4,5,6], the most localised of all Australian cycad genera. *Bowenia* contains two described species, disjunctively distributed in the Wet Tropics of northern Queensland (*B. spectabilis*) and in the Byfield district of central-eastern Queensland (*B. serrulata*) [7,8]. Both are listed as species of Least Concern in the IUCN categories [9,10]. The two currently named species of *Miltotranes* weevils are tightly associated with these *Bowenia* species, *M. prosternalis* (Lea, 1929) with *B. spectabilis* and *M. subopacus* (Lea, 1929) with *B. serrulata*, in that their larvae develop in the male cones of only these cycad species and that the adults pollinate their respective hosts. During the annual coning period, *Miltotranes* adults congregate and mate on the male cones and the females then oviposit in the microsporophylls, and the larvae feed on the mesophyll tissue of the male cones. Upon maturity, the larvae leave the disintegrating cones and pupate in surrounding substrates. Mature larvae and pupae reared under moist and unventilated conditions in the laboratory appeared stressed and quickly died but successfully eclosed under drier and ventilated conditions, which suggests that larval or pupal diapause in the following rainy season is improbable. It appears that next-generation adults eclose by the end of the coning period and estivate until the next coning season [6]. Although the mechanism of pollination and traits responsible for maintaining pollinator specificity in *Bowenia* cycads remain unclear, adult weevils dusted with *Bowenia* pollen have been collected from receptive female cones, indicating their potential of pollinating the female cones [1,2,3,4,5,6]. In pollination experiments, Wilson [4,5] found no difference in fertilisation rate in female cones shielded from wind- and waterborne pollen (but not from weevils), confirming that entomophily is the major pollination mechanism also in *Bowenia* cycads. Oberprieler [11] briefly discussed the evolution of cycad pollination in the *Tranes* group and suggested that the association of *Miltotranes* with *Bowenia* may be due to a secondary colonisation of this cycad genus by the *Tranes* group.

Our taxonomic study of the cycad-associated weevils in Australia has revealed the existence of another, undescribed species of *Miltotranes* in the far north of Queensland, known only from the poorly studied population of *B. spectabilis* in the McIlwraith Range. In this paper we describe this new species and provide an identification key to the three species of *Miltotranes*, together with diagnoses and redescriptions of the previously described species, photographs of adults, larvae and genitalia and a distribution map. A lectotype for the name *Tranes prosternalis* Lea, 1929 is also designated. Furthermore, the taxonomic status of some local populations of *Bowenia* is discussed based on our taxonomic works of *Miltotranes*. The present study thus provides new insights into cycad systematics and conservation from an entomological perspective.

## 2. Materials and Methods

### 2.1. Specimen Depositories

Examined material is housed in the following collections:

ANIC—Australian National Insect Collection, CSIRO, Canberra, ACT, Australia;

QDPI—Queensland Department of Primary Industries, Brisbane, QLD, Australia;

QMBA—Queensland Museum, Brisbane, QLD, Australia;

SAMA—South Australian Museum, Adelaide, SA, Australia.

### 2.2. Specimen Preparation and Photography, Measurements and Terminology

The method of specimen preparation and measurements follow Hsiao and Oberprieler [12]. Photographs were mainly taken using a Leica DFC500 camera mounted on a Leica M205C stereomicroscope, except that photographs of the dorsal habitus of *M. prosternalis* were taken using a Dun Inc. BK Lab Plus system. Images taken at different focus planes were then aligned and stacked in the software program Leica Application Suite (LAS) V4.9 or Helicon Focus (only for dorsal habitus of *M. prosternalis*) and edited with the software program Photoshop CS6. The complete set of unedited photographs of new taxa and those for character coding and comparison are available in the Zenodo archive under the doi: 10.5281/zenodo.6451541. Morphological terminology follows Oberprieler et al. [13]. Label data of type specimens are cited verbatim, with a double slash (//) denoting data from different labels and a single one (/) those on different lines on a label.

### 2.3. Distribution Maps

Locality data from specimen labels were converted into standard GPS format (decimal degree) using Google Maps. These data were imported into ‘GPS visualizer’ (www.gpsvisualizer.com, accessed on 2 May 2022) using the ‘JPEG map’ option. The map was created using the ‘OpenStreetMap (Mundialis)’ background.

## 3. Results

### 3.1. Key to the Adults of Miltotranes Zimmerman, 1994

**Figure 1:**
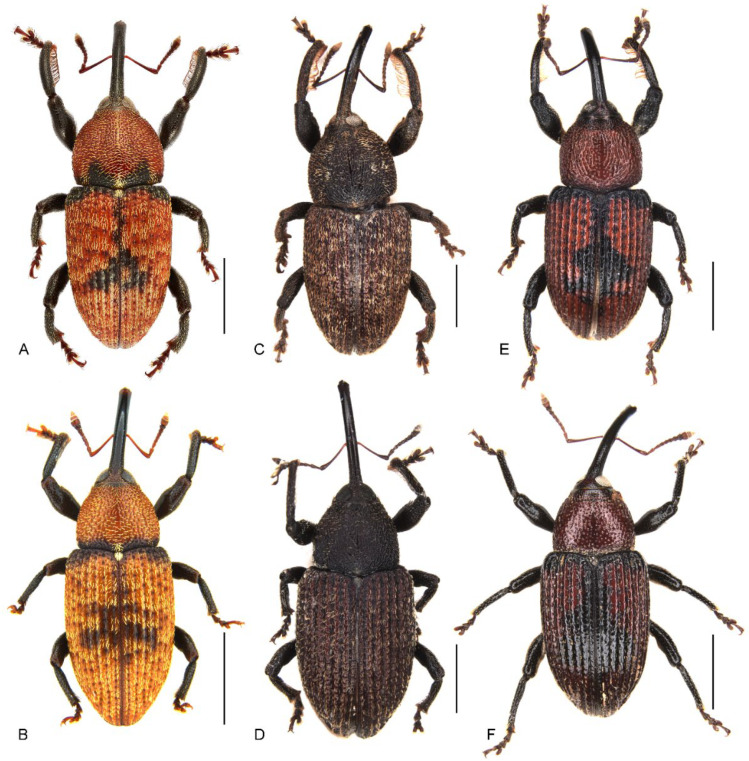
Habitus of *Miltotranes* adults, dorsal view: (**A**) *M. prosternalis*, male; (**B**) ditto, female; (**C**) *M. subopacus*, male; (**D**) ditto, female; (**E**) *M. wilsoni* sp. n., holotype; (**F**) ditto, female, paratype. Scale bars: 2.0 mm.

**Figure 2:**
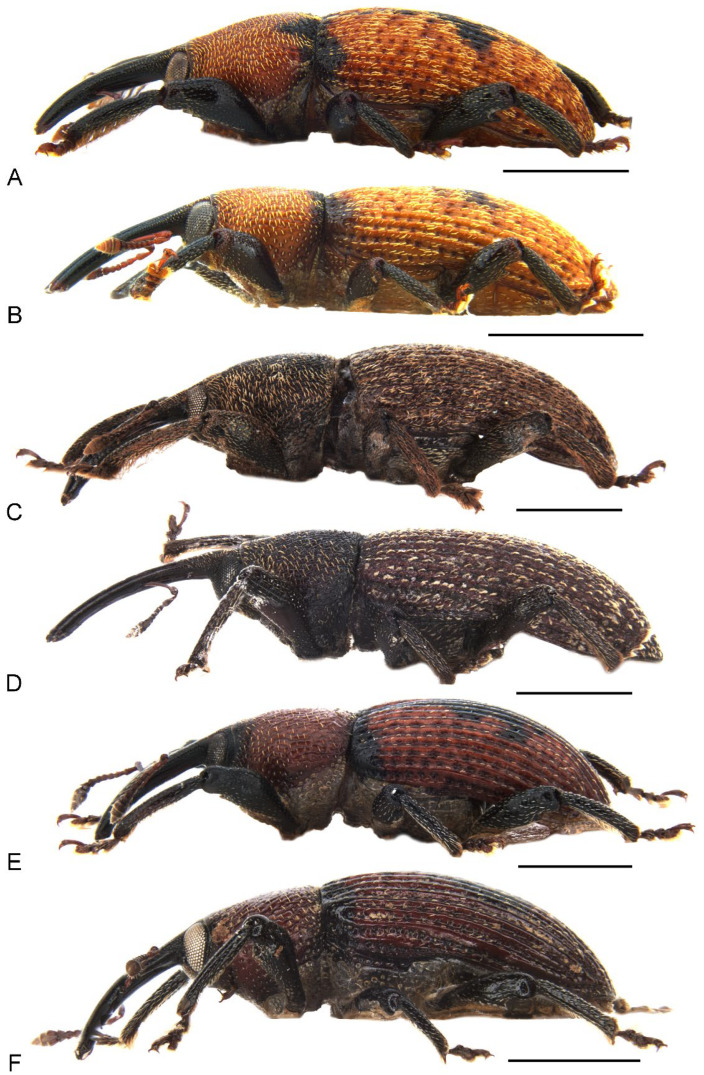
Habitus of *Miltotranes* adults, lateral view: (**A**) *M. prosternalis*, male; (**B**) ditto, female; (**C**) *M. subopacus*, male; (**D**) ditto, female; (**E**) *M. wilsoni* sp. n., holotype; (**F**) ditto, female, paratype. Scale bars: 2.0 mm.

1. Body uniformly dark brown (Figure 1C,D); antennae inserted in middle of rostrum in male, slightly behind middle in female (Figure 1C,D and Figure 2C,D); abdominal ventrite 5 in female distinctly depressed posteriorly (Figure 7B); distribution: Byfield district of central-eastern Queensland………………………………………………………………*M. subopacus*
— Body orange to dark red with large black macula in middle of elytra (Figure 1A,B,E,F); antennae inserted slightly before middle in male, in middle in female ( Figure 1A,B,E,F and Figure 2A,B,E,F); abdominal ventrite 5 in female flat, without depression (Figure 7A,C); distribution: northern Queensland……………………………………………………………22. Pronotum with a pair of triangular black marks at posterior margin and black elytral macula irregular, broken and mosaic (Figure 1A,B); pronotal and elytral setae densely distributed, clustered in parts to somewhat obscure derm (Figure 1A,B and Figure 4A); pronotum ca. 0.8–0.9× as broad as elytra at humeri (Figure 1A,B); protibiae ca. 6.0× longer than wide (Figure 6A); distribution: Wet Tropics of Queensland ……………*M. prosternalis*— Pronotum unicolorous, without black marks, and black elytral macula entire (Figure 1E,F); pronotal and elytral setae sparsely distributed (Figure 1E,F and Figure 4C); pronotum ca. 0.7–0.8× as broad as elytra at humeri (Figure 1E,F); protibiae ca. 7.5× longer than wide (Figure 6C); distribution: McIlwraith Range of Cape York Peninsula of Queensland ……………………………………………………………………………………………*M. wilsoni*

### 3.2. Redescription of Genus

#### ***Miltotranes*** Zimmerman, 1994

(Figure 1, Figure 2, Figure 3, Figure 4, Figure 5, Figure 6, Figure 7, Figure 8, Figure 9, Figure 10, Figure 11 and Figure 12)

*Miltotranes* Zimmerman, 1994: 695 [14].—Oberprieler, 1995a: 306, 329 [15]; 1995b: 338 [16]; 2004: 174, 183 [11] (classification, host associations); Alonso-Zarazaga & Lyal, 1999: 210 [17] (catalogue); Hill & Osborne, 2001: 4 [7] (host associations); Jones, 2002: 52 [8] (host associations); Wilson, 2001: 21 [2]; 2002a: 13, 16 [3]; 2002b: 440 [5]; 2004: 53 [5] (pollination, habits); Oberprieler & Caldara, 2012: 57 [18] (classification, habits); Lyal, 2014: 560 [19] (classification); Pullen et al., 2014: 289 [20] (catalogue); Anderson et al., 2018: 2 [21] (classification); Legalov, 2018: 345 [22] (key, catalogue, classification); Hsiao & Oberprieler, 2020a: 369 [6] (habits); 2020b: 677 [12] (classification); Toon et al., 2020: 1044 [23] (host associations).

Type species, by original designation: *Tranes prosternalis* Lea, 1929.

##### Diagnosis

**Figure 3:**
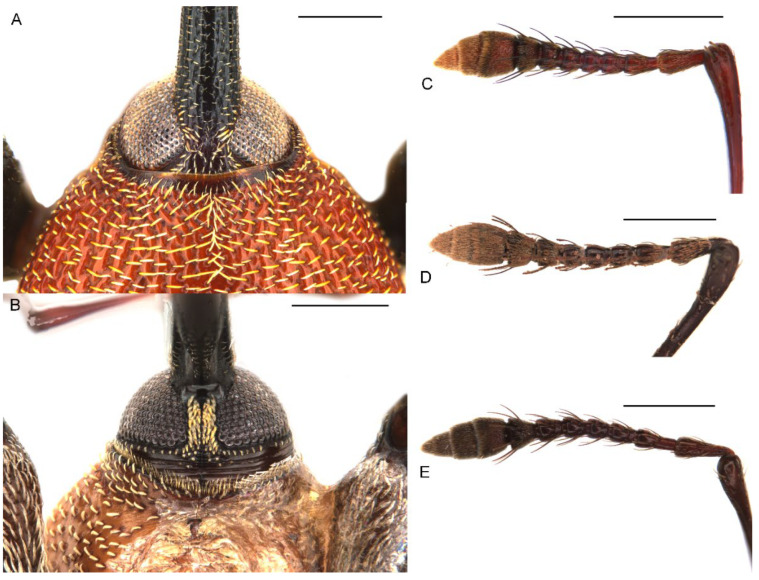
Diagnostic external characters of *Miltotranes*: (**A**) *M. prosternalis*, head and base of rostrum, dorsal view; (**B**) ditto, ventral view; (**C**) *M. prosternalis*, right antenna, dorsal view; (**D**) *M. subopacus*, ditto; (**E**) *M.* *wilsoni* sp. n., ditto. Scale bars: 0.5 mm.

*Miltotranes* can be distinguished from other genera of the *Tranes* group by the following characters (states of genera of *Tranes* group in parentheses): small-sized body, length ≤ 8.0 mm (larger, length usually > 8.0 mm in other genera except for *Tranes lyterioides* (Pascoe, 1875) and its closely related species); dark brown to reddish brown body (Figure 1 and Figure 2) (completely black in *Demyrsus* Pascoe, 1872, *Siraton* Hustache, 1934, *Paratranes* Zimmerman, 1994 and *Howeotranes* Zimmerman, 1994); interocular distance ca. 0.6× basal width of rostrum in dorsal view (Figure 3A) (as wide as basal width of rostrum in *Demyrsus*, *Siraton* and *Tranes*, and ca. 0.2× basal width of rostrum in *Howeotranes*); funicle segments 7 distinctly separated from club (Figure 3C–E) (closely approximated to basal club segment in *Paratranes* and *Howeotranes*); clubs distinctly shorter than funicles (as long as funicles in *Demyrsus*); prothorax with anterior margins without ventral emargination (Figure 3B) (distinctly emarginate ventrally in *Demyrsus* and *Siraton*); pronotum with surface punctorugulose (punctate in *Siraton*, *Paratranes*, *Howeotranes* and *Tranes*); prosternum prominently protuberant in male (not so in *Demyrsus*, *Siraton*, *Paratranes* and *Howeotranes*); procoxal cavities separated (confluent in *Paratranes* and *Howeotranes*); metanepisterna without sclerolepidia (with sclerolepidia in *Demyrsus* and *Siraton*); femora not sulcate (sulcate beneath in *Paratranes*), thicker in male (Figure 1) (not so in other genera); protibiae thicker, more curved and with inner setal brush in male (Figure 6A–C) (thinner and straighter in other genera and without brush in *Demyrsus*, *Siraton*, *Paratranes* and *Howeotranes*); meso- and metatibiae with distal setal comb restricted to apical margin (Figure 6D–F) (extending to middle or slightly before middle of tibiae in *Paratranes* and *Howeotranes*).

##### Redescription

**Figure 4:**
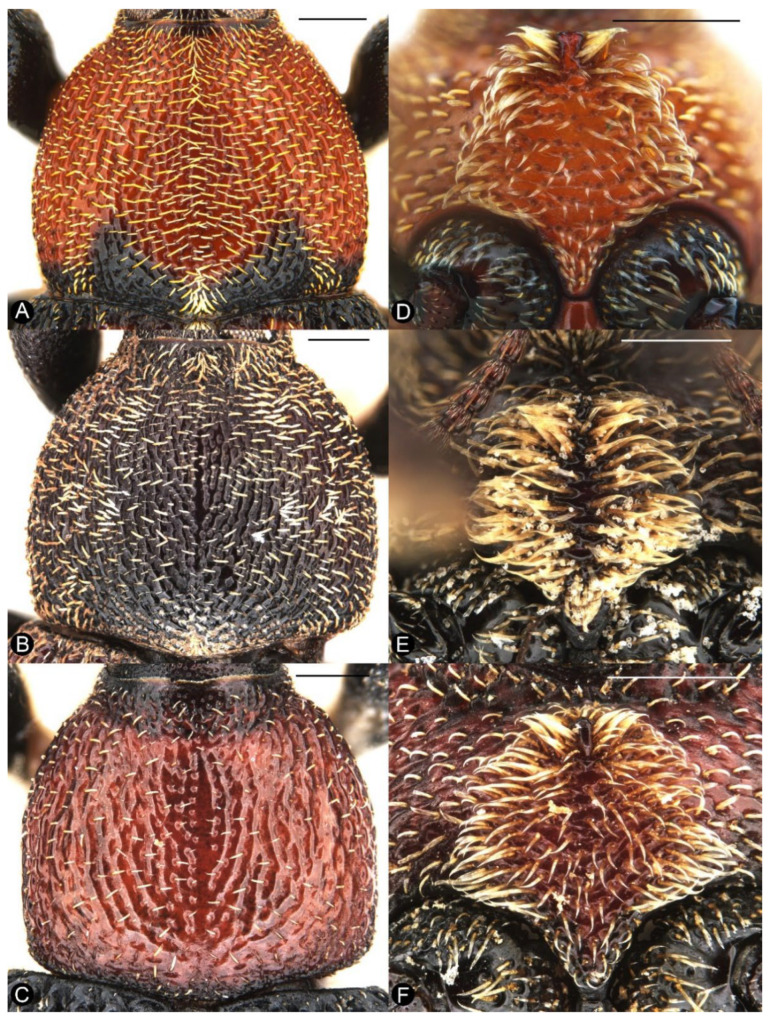
Diagnostic external characters of *Miltotranes*: (**A**) *M. prosternalis*, pronotum, dorsal view; (**B**) *M. subopacus*, ditto; (**C**) *M. wilsoni* sp. n., ditto; (**D**) *M. prosternalis*, prosternal elevated, erect-setose process of male; (**E**) *M. subopacus*, ditto; (**F**) *M. wilsoni* sp. n., ditto. Scale bars: 0.5 mm.

**Figure 5:**
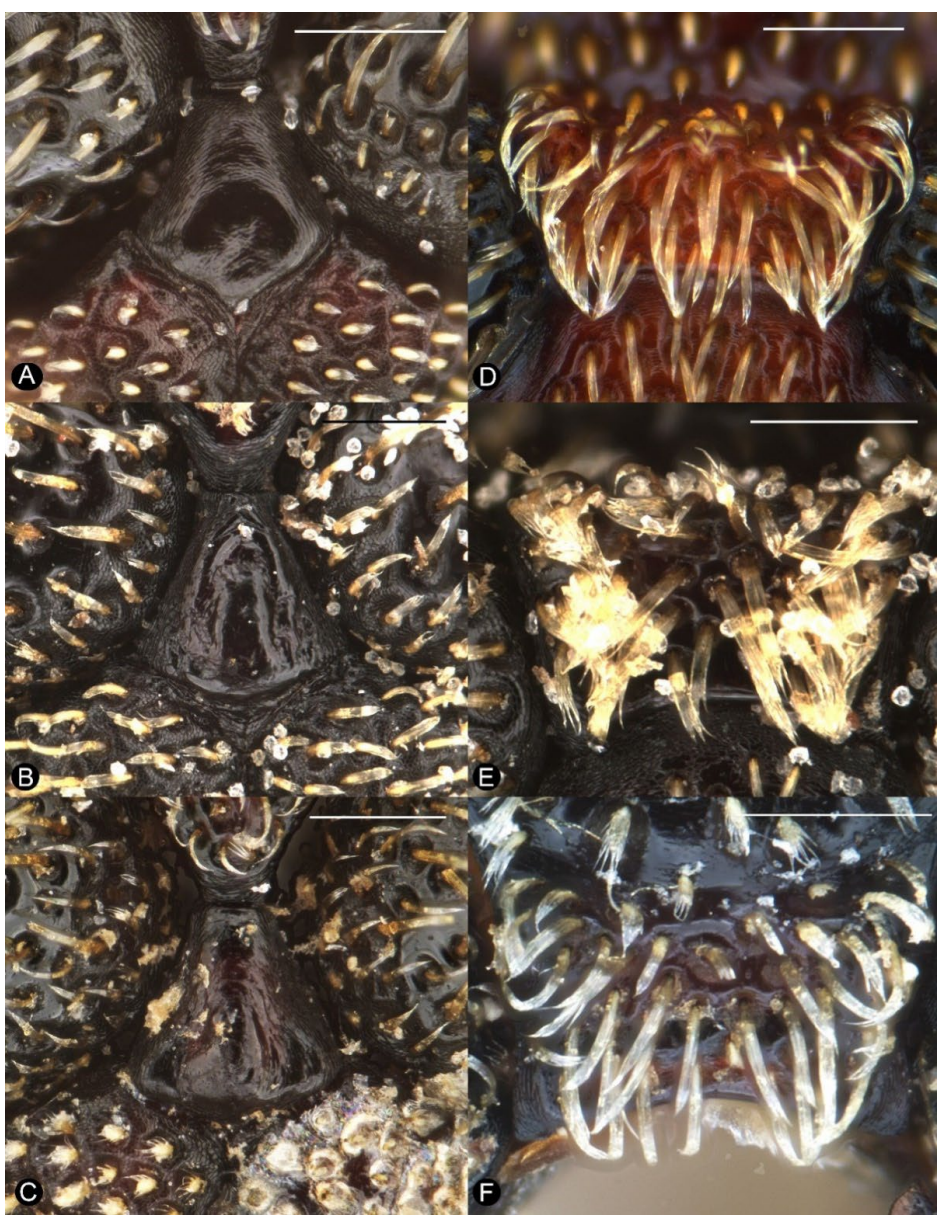
Diagnostic external characters of *Miltotranes*: (**A**) *M. prosternalis*, prosternellum; (**B**) *M. subopacus*, ditto; (**C**) *M. wilsoni* sp. n., ditto; (**D***) M. prosternalis*, intermesocoxal process; (**E**) *M. subopacus*, ditto; (**F**) *M. wilsoni* sp. n., ditto. Scale bars: 0.2 mm.

**Figure 6:**
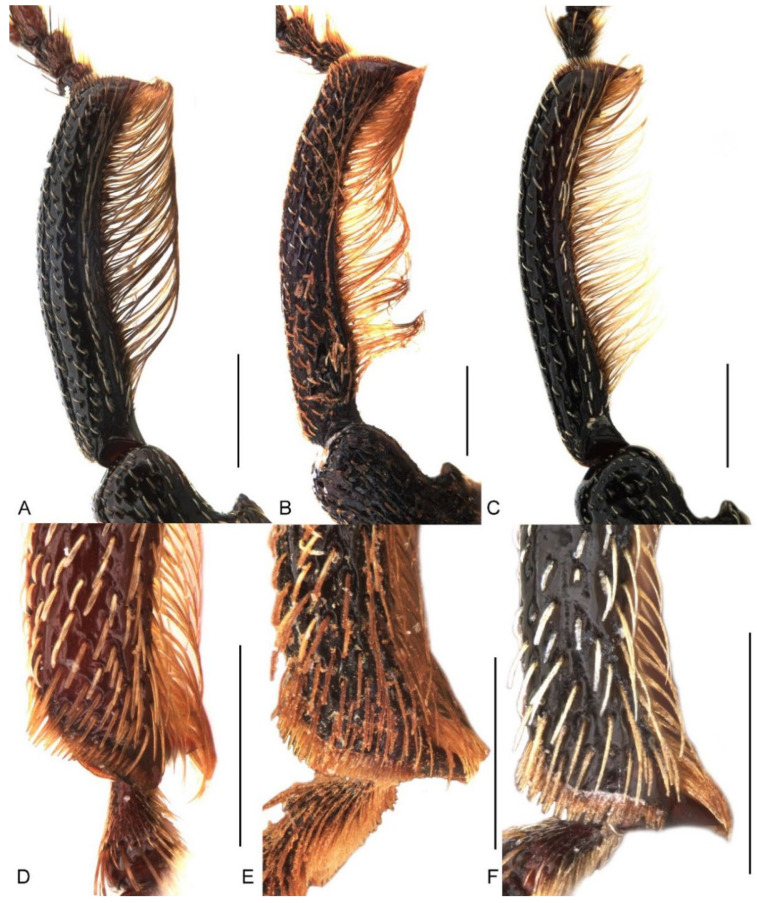
Diagnostic external characters of *Miltotranes*: (**A**) *M. prosternalis*, left protibia of male, dorsal view; (**B**) *M. subopacus*, ditto; (**C**) *M. wilsoni* sp. n., ditto; (**D**) *M. prosternalis*, left mesotibia of male, dorsal view; (**E**) *M. subopacus*, ditto; (**F**) *M. wilsoni* sp. n., ditto. Scale bars: 0.5 mm.

**Figure 7:**
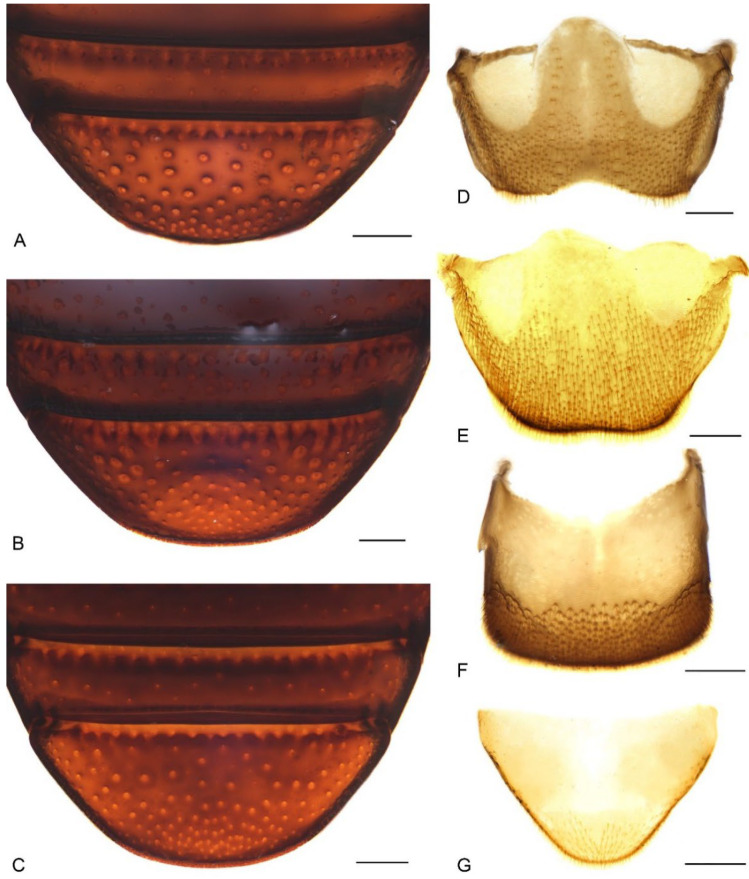
Diagnostic external characters and terminalia of *Miltotranes*: (**A**) *M. prosternalis*, abdominal ventrite 5 of female, ventral view; (**B**) *M. subopacus*, ditto; (**C**) *M. wilsoni* sp. n., ditto; (**D**) *M. prosternalis*, tergite VII of male, dorsal view; (**E**) ditto, female; (**F**) ditto, tergite VIII of male, dorsal view; (**G**) ditto, female. Scale bars: 0.2 mm.

**Figure 8:**
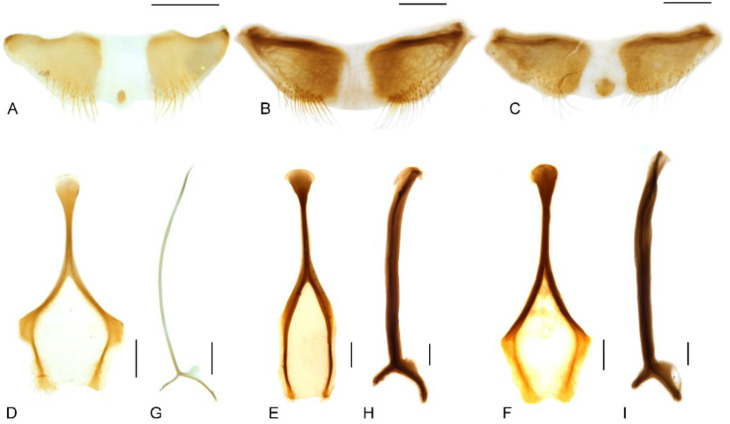
Diagnostic characters of terminalia of *Miltotranes*: (**A**) *M. prosternalis*, abdominal sternite VIII of male, dorsal view; (**B**) *M. subopacus*, ditto; (**C**) *M. wilsoni* sp. n., ditto; (**D**) *M. prosternalis*, sternite VIII of female, dorsal view; (**E**) *M. subopacus*, ditto; (**F**) *M. wilsoni* sp. n., ditto; (**G**) *M. prosternalis*, spiculum gastrale, dorsal view; (**H**) *M. subopacus*, ditto; (**I**) *M. wilsoni* sp. n., ditto. Scale bars: 0.2 mm.

**Figure 9:**
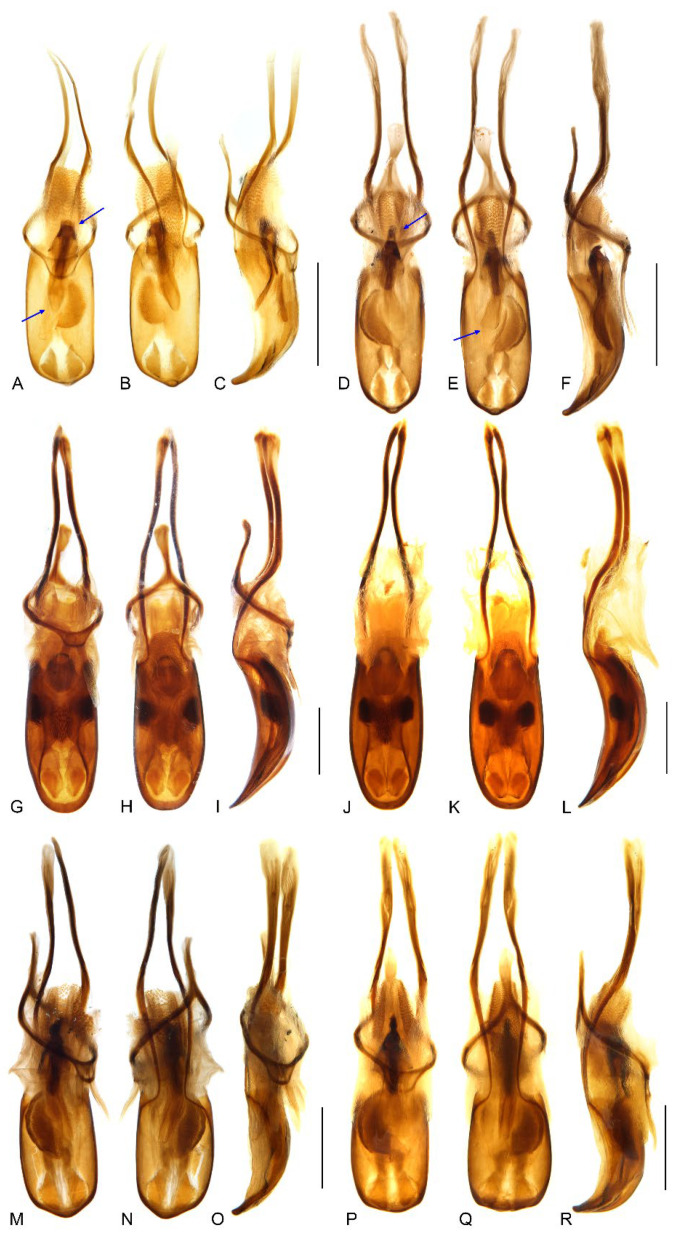
Male genitalia of *Miltotranes*: (**A**) *M. prosternalis*, north of Cairns (Cape Tribulation), dorsal view; (**B**) ditto, ventral view; (**C**) ditto, lateral view; (**D**) *M. prosternalis*, south of Cairns (Mission Beach), dorsal view; (**E**) ditto, ventral view; (**F**) ditto, lateral view; (**G**) *M. subopacus*, dorsal view; (**H**) ditto, ventral view; (**I**) ditto, lateral view; (**J**) ditto, with parameroid lobes removed, ventral view; (**K**) ditto, ventral view; (**L**) ditto, lateral view; (**M**) *M. wilsoni* sp. n., holotype, dorsal view; (**N**) ditto, ventral view; (**O**) ditto, lateral view; (**P**) ditto, paratype, dorsal view; (**Q**) ditto, ventral view; (**R**) ditto, lateral view. Fine differences in endophallus of *M. prosternalis* from north and south of Cairns indicated by blue arrows. Scale bars: 0.5 mm.

**Figure 10:**
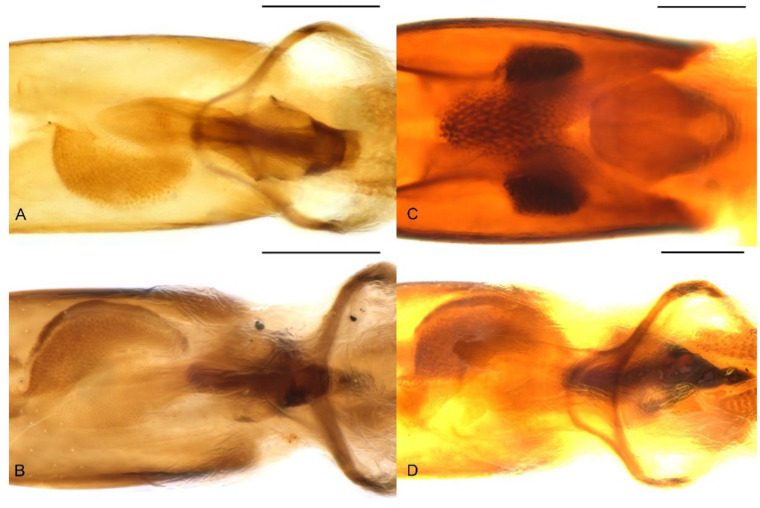
Endophallus of *Miltotranes*: (**A**) *M. prosternalis*, north of Cairns (Cape Tribulation), dorsal view; (**B**) ditto, south of Cairns (Mission Beach), dorsal view; (**C**) *M. subopacus*, dorsal view; (**D***) M. wilsoni* sp. n., dorsal view. Scale bars: 0.2 mm.

**Figure 11:**
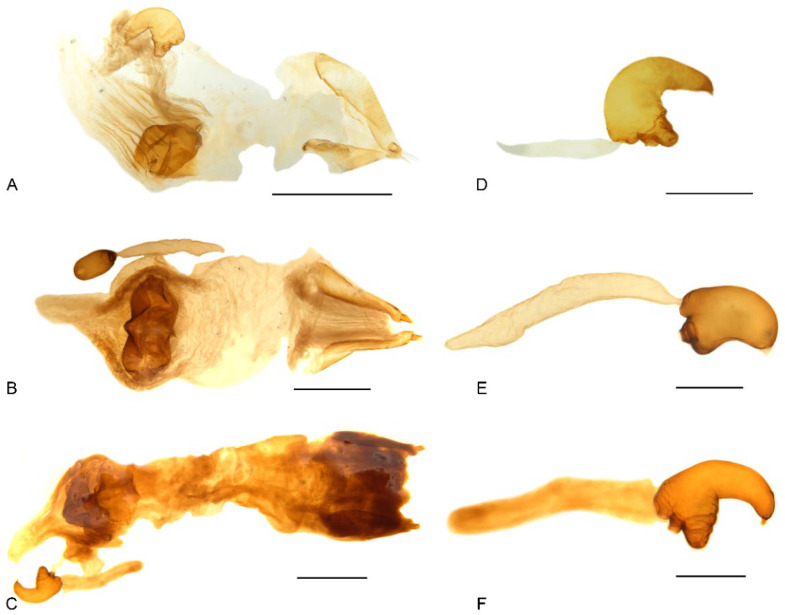
Female genital structures of *Miltotranes*: (**A**) *M. prosternalis*, genitalia; (**B**) *M. subopacus*, ditto; (**C**) *M. wilsoni* sp. n., ditto; (**D**) *M. prosternalis*, spermatheca with gland; (**E**) *M. subopacus*, ditto; (**F**) *M. wilsoni* sp. n., ditto. Scale bars: (**A**,**B**) 0.5 mm; (**D**–**F**) 0.2 mm.

Size small (length ca. 5.5–8.0 mm). Body and legs black to reddish brown, covered with yellowish, coarse, sublanceolate and subsquamiform setae on dorsum, setae regularly distributed or condensed in clusters in some parts (Figure 1). Rostrum moderately long, longer in female (Figure 2). Eyes dorsally well separated, interocular distance ca. 0.5× greatest diameter of eye in dorsal view (Figure 3A); ventrally very narrowly separated, interocular distance ca. 0.4× greatest diameter of eye in ventral view (Figure 3B); forehead slightly narrower than basal width of rostrum (Figure 3A). Antennae inserted in middle or slightly before middle of rostrum in male (Figure 2A,C,E), in middle or slightly behind middle in female (Figure 2B,D,F); funicles distinctly 7-segmented, segments 1 and 2 longer than remaining segments, 2 slightly longer than or as long as apical width of scape and slightly shorter than segments 3 + 4 (Figure 3C–E); clubs stout and short, distinctly shorter than funicles, ca. 0.3–0.4× length of funicle, 4-segmented, with small conical apical segment. Pronotum ca. 0.7–0.9× as broad as elytra at humeri, sides weakly to moderately arcuate (Figure 4A–C); surface punctorugulose, punctures separate on disc but confluent and vague laterally. Prothorax without ocular lobes; prosternum distinctly and densely punctate, with large, elevated, erect-setose protuberance in male (Figure 4D–F); procoxal cavities narrowly separated; prosternellum elongate, widened posteriorly (Figure 5A–C); intermesocoxal process trapezoidal, densely setose (Figure 5D–F); metanepisterna without sclerolepidia. Elytra oval, jointly ca. 0.65× as broad as long, sides narrowing apicad (Figure 1); surface roundly convex (Figure 2). Femora thicker in male; protibiae thicker, more curved and with well-developed tibial brush in male (Figure 6A–C); meso- and metatibiae with distal setal comb restricted to apical margin, not ascending on posterior edge (Figure 6D–F), metatibiae with dorso-apical corner rounded. Terminalia: tergite VII of male transverse (Figure 7D), moderately emarginate medially, of female subtrapezoidal (Figure 7E), with posterior margin subtruncate, anterior margin ca. 1.7–1.9× wider than posterior margin; tergite VIII of male subquadrate (Figure 7F), with posterior margin subtruncate, of female subtriangular (Figure 7G), with rounded apex, distinctly narrowed apicad, anterior margin ca. 3.3–3.9× wider than posterior margin; sternite VIII of male narrowly subtrapezoidal (Figure 8A–C), largely sclerotised but medially membranous, membrane with small circular sclerite medially in *M. prosternalis* and *M. wilsoni*, of female with sclerotised parts of apical lobes slender, linear, laterally slightly curved or abruptly angled (Figure 8D–F); spiculum gastrale asymmetrical, widely concave apically (Figure 8G–I); tegmen with complete ring, manubrium as long as parameroid lobes (Figure 9); penis subparallel (Figure 9), strongly sclerotised, forming a median groove narrowing apicad and roundly open in apical third, apical margin rounded or obtusely pointed, body shorter than temones (ca. 1.2–1.3×); endophallus membranous, long, extending anteriorly of body of penis, basally with a membranous sleeve of dense denticles, medially with a complex symmetrical sclerite (Figure 10) composed of rounded or rhombic ventral part and flatly spoon- or plier-shaped dorsal part, distally with asymmetrical or symmetrical patch of denticles; ovipositor short, nearly as long as wide (Figure 11), gonocoxites basally broad, narrowing apicad, styli subapical, broad, apically truncate, with few long setae.

### 3.3. Redescription of Species

#### 3.3.1. ***Miltotranes prosternalis*** (Lea, 1929)

(Figure 1A,B, Figure 2A,B, Figure 3C, Figure 4A,D, Figure 5A,D, Figure 6A,D, Figure 7A, Figure 8A,D,G, Figure 9A–F, Figure 10A,B, Figure 11A,D, Figure 13A and Figure 14)

*Tranes prosternalis* Lea, 1929: 538 [24]—Schenkling & Marshall, 1936: 1 [25]; Zimmerman, 1994: 696 [14]; Oberprieler, 1995a: 306 [15]; Alonso-Zarazaga & Lyal, 1999: 210 [17]; Pullen et al., 2014: 289 [20].

*Miltotranes prosternalis* (Lea)—Zimmerman, 1994: 696 [14]; Oberprieler, 1995a: 307, 329 [15]; 1995b: 338 [16]; Jones, 2002: 52 [8]; Wilson, 2002a: 13, 16 [3]; 2002b: 440 [4]; 2004: 57 [5]; Pullen et al., 2014: 289 [20]; Hsiao & Oberprieler, 2020a: 369 [6]; Toon et al., 2020: 1044 [23].

Type locality: Endeavour River, Queensland, Australia.

##### Material Examined

Types. Lectotype (Figure 13A), ♂: “*prosternalis* / Lea, TYPE / Endeavour R // Specimen / figured / ECZ // *Tranes* 13580 / *prosternalis* / Lea / Queensland. / TYPE // LECTOTYPE / *Tranes prosternalis* / Lea, 1929 / des. Hsiao & Oberprieler, 2022” (SAMA). Paralectotype, ♂: “Endeavour / River. // *prosternalis* / Lea, Co-type // *Tranes* 17016 / *prosternalis* Lea / Queensland. / Cotype // PARALECTOTYPE / *Tranes prosternalis* / Lea, 1929 / des. Hsiao & Oberprieler, 2022” (SAMA).

Other Material. Queensland: (no data) (1 ♂, ANIC); Cairns (no date or collector name), *Tranes prosternalis* c.w.t., E.C. Zimmerman (1 ♀, ANIC); Endeavour (no date or collector name) (1 ♂, 1 ♀, ANIC); Mission Beach, NQ, 10.XI.1965, G. Monteith leg., *Tranes prosternalis* c.w.t., E.C. Zimmerman (1 ♂, ANIC); Cardstone, XI.1966, K. H. leg. (1 ♂, ANIC); Townsville, XI.1966, E. Wollaston leg. (2 ♂, 6 ♀, ANIC); W. shore of L. Tinaroo, 2500, NQ, 7.XI.1966, E. Britton leg. (1 ♀, ANIC); 8 mi., E. of Ravenshoe, Maalan, 19.XI.1968, R.J. Elder leg. (1 ♀, ANIC); same locality, 26.XI.1968 (no collector name) (1 ♂, ANIC); Base Cableway, Mt. Bellenden-Ker, 80 m, 17.16S 145.54E, 25.X.1981, E.D. Edwards leg. (1 ♀, ANIC); 15 km WNW of South Johnstone, N. QLD, 24.XII.1985, Fay & Halfpapp leg. (1 ♀, QDPI); same locality and collector, X.1987 (1 ♀, QDPI); same locality and collector, XI.1987 (1 ♀, QDPI); Tully R. Xing, 10 km S. Koombooloomba Dam, 750 m, N. QLD, 8.XII.1989–4.I.1990, Monteith, Thompson & Janetski leg. (1 ♀, QMBA); South Johnstone R. S., 11.X.–29.XI 1990, K.H. Halfpapp leg. (3 ♂, 1 ♀, QDPI); Garradunga, 20.XII.1990, J. Hasenpusch leg. (1 ♀, ANIC); same locality and collector, 1.XI.1991–15.II.1992 (2 ♂, ANIC); 17.28S 146.01E, 2km E of Garradunga, XI.1992, J. Hasenpusch leg. (1 ♂, 3 ♀, ANIC); 17.32S 146.01E, Innisfail, XI.1992 (no collector name) (1 ♀, ANIC); Garradunga, 10.XI.1992, J. Hasenpusch leg. (1 ♀, QDPI); Innisfail, Garradunga, 10.XII.1992, Hasenpusch leg. (1 ♂, QDPI); Stone Creek, Garadunga, N. QLD, 20–25.I.1994, P. Hasenpusch leg. (1 ♂, QDPI); Josephine Creek, 16.IX.1994, G. Wilson leg. (4 ♂, 5 ♀, 3 larvae, ANIC); Kuranda, 21.XI.1994, G. Wilson leg. (1 ♂, 4 ♀, ANIC); Tinaroo, 22.XI.1994, G. Wilson leg. (11 ♂, 9 ♀, ANIC); 17.37S 145.34E, 1000 m, BS3 Massey Ck., 1.XII.1994–3.I.1995, P. Zborowski leg. (1 ♂, ANIC); 17.35S 145.35E, Maalan SF on Hwy, 850 m, NEQ, 25.XI.1994–10.I.1995, Monteith & Hasenpusch leg. (1 ♂, QMBA); 17.28S 146.01E, Stone Ck, 100 m, NEQ, 1.III.–20.V.1995, J. Hasenpusch leg. (3 ♂, QMBA); same locality and collector, 1.X.–1.XI.1995 (1 ♂, 1 ♀, QMBA); South Johnstone, 29.XI.1997–14.I.1998, K.H. Halfpapp leg. (1 ♀, ANIC); 17.457° S 146.020° E, Polly Ck, Garradunga, 9.XI.2009, J. Hasenpusch leg. (2 ♀, ANIC); 17.459° S 146.021° E, Polly Ck, Garradunga, 6–17.XII.2009, J. Hasenpusch leg. (1 ♀, ANIC); 17°23.136′ S 145°59.596′ E, Bramston Beach Rd. on Powerlines Rd (under powerlines), 93m, 4.XI.2015, D.C.F. Rentz & B. Richardson leg. (1 ♂, ANIC); −16.2374737 145.4275167, Near Daintree Discovery Centre, 27.X.2019, Z. Liu leg. (1 ♂, ANIC); same locality, 27.X.2019, mature larvae eclosed on 10–16.XII.2019, Y. Hsiao & Y. Li leg. (8 ♂, 21 ♀, ANIC); −16.2375316 145.4278302, Tulip Oak Rd, Turn off Cape Tribulation Rd, Cow Bay, 27.X.2019, Z. Liu leg. (1 ♂, ANIC); −16.2375043 145.4287016, 14 Tulip Oak Rd, Cow Bay, 27.X.2019, Y. Hsiao & Y. Li leg. (1 ♂, 2 ♀, ANIC); Marrdja Botanical Walk, 27.X.2019, Y. Hsiao leg. (2 ♂, 1 ♀, 20 larvae, ANIC); −16.0871718 145.4642166, Near PK**’**s Jungle Village, Cape Tribulation, 27.X.2019, H. Escalona leg. (1 ♂, ANIC); −16.2259692, 145.4214418, Near Floravilla Ice Cream Factory, 335 Cape Tribulation Rd, Cow Bay, 27.X.2019, Z. Liu leg. (1 ♀, ANIC); −16.103933 145.449165, Daintree Rainforest Observatory, JCU, 48m, 27.X.2019, mature larvae eclosed on 12.XII.2019, H. Escalona leg. (7 ♂, 3 ♀, ANIC).

##### Diagnosis

Adults of this species are externally extremely similar to *M. wilsoni* but distinguishable from it by the following characters (states of *M. wilsoni* in parentheses): pronotum with a pair of triangular black marks on posterior margin and elytra with interval 1 alternating black and orange along basal half and a broken and mosaic median black macula (Figure 1A,B) (pronotum unicolorous, without black marks, and elytra with interval 1 uniformly black along basal half and an entire median black macula; Figure 1E,F); vestiture on pronotum and elytra longer, dense, clustered in parts to somewhat obscure derm (Figure 1A,B and Figure 4A) (shorter, sparsely distributed; Figure 1E,F and Figure 4C); pronotum broader, ca. 0.8–0.9× as broad as elytra, lateral margins distinctly rounded in male (Figure 1A,B and Figure 4A) (narrower, ca. 0.7–0.8× as broad as elytra, lateral margins weakly rounded in male; Figure 1E,F and Figure 4C); protibiae thicker in male, ca. 6.0× as long as wide (Figure 6A) (more elongate and slender in male, ca. 7.5× as wide; Figure 6C); penis more elongate, ca. 1.9–2.1× longer than wide (Figure 9A,B,D,E) (penis thicker, ca. 1.6–1.7× longer than wide; Figure 9M,N,P,Q).

##### Redescription

Shape and size. Body broadly oval (Figure 1A,B), length 5.4–6.4 mm in both sexes, width ca. 0.4–0.5× length, moderately convex in lateral view (Figure 2A,B).

Colour and vestiture. Head dark red to black, antennae reddish brown, thorax orange to dark red, pronotum with a pair of triangular black marks at base, elytra orange to dark red, with black anterior margin and anterior half of elytral intervals 1 and an irregular black mark medially, abdomen dark red, coxae, trochanters, femora and tibiae black, tarsi reddish brown, semilustrous (Figure 1A,B); body and legs covered with coarse, sublanceolate and subsquamiform, yellowish setae, clustered in some parts to somewhat obscure derm, especially on pronotum, prosternal elevated process in male, scutellar shield, intermesocoxal process in male and elytra, setae longer on pronotum, prosternal elevated process in male, intermesocoxal process in male and elytra, setae denser in margins of prosternal elevated process in male.

Head. Rostrum: moderately long, longer in female (ca. 1.3× longer than pronotum in male, 1.5× in female), robust (ca. 6.0× as long as wide in male, 6.5× in female), downcurved, dorsoventrally flattened, slightly broadened apically in dorsal view, coarsely punctate dorsally, punctures slightly smaller in distal half, proximal half with paired dorsomedian and dorsolateral carinae, the latter lower than the former. Eyes: subcircular in outline, slightly convex but not protruding in dorsal view (Figure 1A,B). Antennae: inserted slightly before middle of rostrum in male (Figure 2A), in middle in female (Figure 2B); scapes not reaching eye; funicles with segment 1 longest, ca. 1.4×, 2.3×, 2.5×, 2.3×, 2.3× and 2.1× longer than segments 2 to 7, respectively; clubs elongate, ca. 1.8× longer than wide, densely and finely pubescent (Figure 3C).

Thorax. Pronotum: roundly trapezoidal, apex ca. 0.5× narrower than base (Figure 4A); anterior margin subtruncate, slightly emarginate medially, posterior margin protruding medially, forming obtuse median lobe, lateral margins mostly rounded but distinctly converging anteriad; disc weakly and evenly convex; surface distinctly punctorugulose, with median longitudinal ridge. Prosternum: with a large, subrhombic, elevated, erect-setose patch in male, with a small process anteriorly (Figure 4D); prosternellum elongate, widened posteriorly (Figure 5A). Mesoventrite: intermesocoxal process trapezoidal, with anterior margin shallowly emarginate and anterior angles protuberant in male (Figure 5D). Scutellar shield: roundly subpentagonal. Elytra: ca. 2.2–2.6 × longer than pronotum, jointly ca. 0.6–0.7× as broad as long, broader than base of pronotum; humeri broadly rounded, slightly protruding; surface uneven, deeply and coarsely punctate in rows, forming distinct striae, interstriae convex. Legs: femora with small ventral subapical tooth; tibiae with premucro smaller than uncus; protibiae stronger and more curved, with well-developed tibial brushes in male (Figure 6A); meso- and metatibiae with distal setal combs short, restricted to apical margin (Figure 6D); tarsi with claws free, divergent. Abdomen: ventrite 5 even, without depression in both male and female (Figure 7A).

Terminalia. Male: sternite VIII subtrapezoidal, sclerotised, apical margin rounded to truncate, apical margin with a small rounded sclerite medially, basal margin strongly sclerotised (Figure 8A); spiculum gastrale widely concave apically, base lightly sclerotised (Figure 8G); tegmen with complete ring, manubrium slightly shorter than parameroid lobes (Figure 9A–F); penis thick (ca. 1.9–2.1× longer than wide), subparallel-sided, distinctly narrowing apicad in apical one fifth, obtusely pointed apically (Figure 9A,B,D,E); endophallus with a complex of sclerites inside penis composed of basal membranous sleeve of denticles, median elongate rhombic sclerite and distal crescent-shaped patch of dense denticles (Figure 10A,B). Female: sternite VIII abruptly angled laterally (Figure 8D); gonocoxites thick, short, apically bluntly rounded (Figure 11A); gonostyli short, conical, bluntly rounded and setose apically; bursa copulatrix without bands of spicules; spermatheca thick, right-angled, gland small, narrower than spermatheca, elongate, narrowing apicad (Figure 11D).

**Figure 12:**
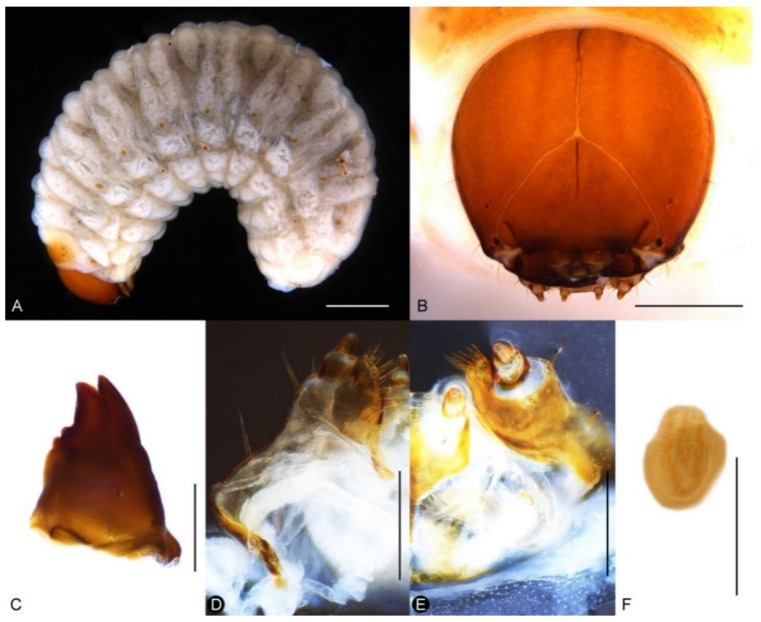
Diagnostic characters of final-instar larva of *Miltotranes prosternalis*: (**A**) habitus, lateral view; (**B**) head, frontal view; (**C**) right mandible, dorsal view; (**D**) maxilla, dorsal view; (**E**) maxilla and labium, ventral view; (**F**) abdominal spiracle II. Scale bars: (**A**) 1.0 mm; (**B**) 0.5 mm; (**D**–**F**) 0.2 mm.

Larva. Body of nearly equal width throughout (Figure 12A), without black spinules on prodorsal fold, setae brown. Head reddish brown, free, without pale lines extending beyond dorso-epicranial setae 1 (Figure 12B); postoccipital condyles present; mandibular setae aligned longitudinally (Figure 12C); head width of last instar ca. 1.1–1.2 mm. Mala of maxillae with 8 thick dorsal setae and 2 ventral setae (Figure 12D,E); postlabium without a basal, pigmented bar. Spiracles with small atrium and long airtubes, airtubes as long as width of peritreme (Figure 12F).

##### Distribution

*Miltotranes prosternalis* occurs in the coastal regions of far northern Queensland, recorded from the Endeavour River at Cooktown in the north to Townsville in the south (Figure 14).

##### Natural History

*Miltotranes prosternalis* is exclusively associated with *Bowenia spectabilis* (except for the McIlwraith Range population, doubtfully regarded as *B. spectabilis*) and is the apparent sole pollinator of its host, developing in the male cones of the plants mainly from October to December. Its habits and interaction with *B. spectabilis* have been summarised by Hsiao & Oberprieler [6].

##### Remarks

**Figure 13:**
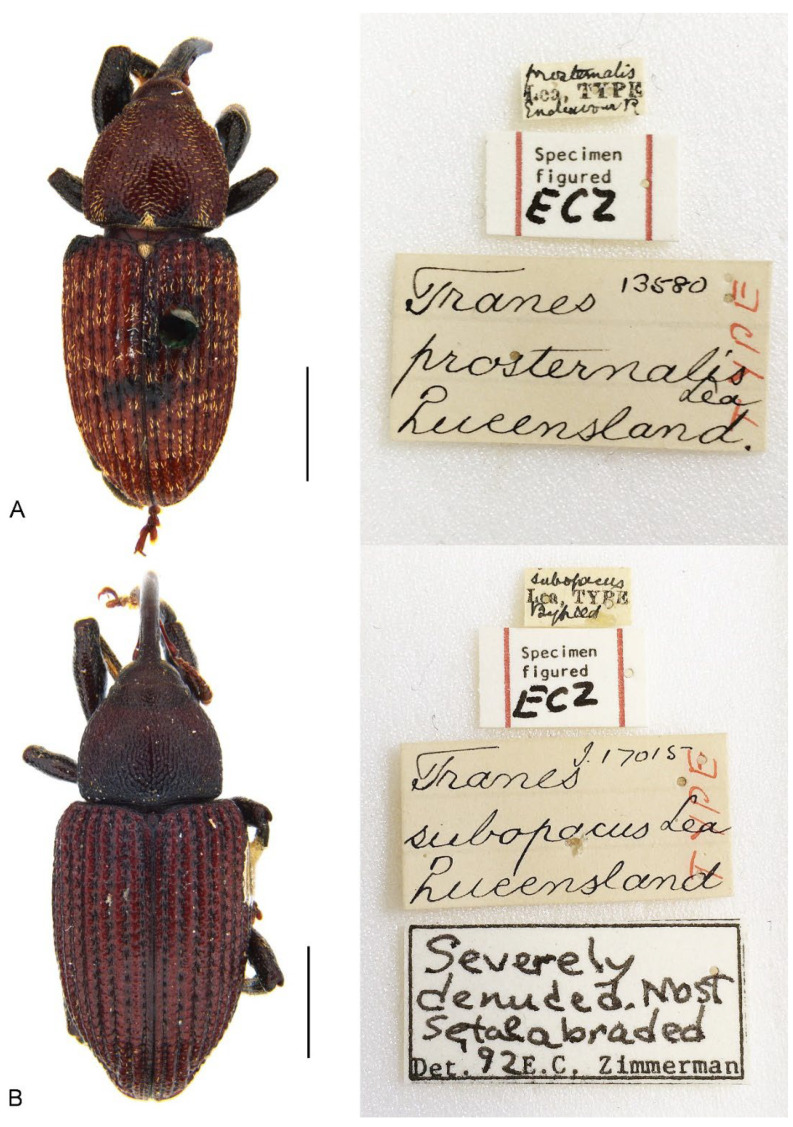
Name-bearing types of *Miltotranes*: (**A**) *Tranes prosternalis* Lea, 1929, lectotype; (**B**) *Tranes subopacus* Lea, 1929, holotype. Scale bar: 2.0 mm.

Lea [24] described *Tranes prosternalis* based on specimens from the Endeavour River in the far north of Queensland. In his description he wrote that he had “… two specimens before me”, indicating that the type series only comprises two specimens, and in his collection (in SAMA) there is a male labelled “Type” and another male labelled “co-type”. However, as he did not designate a primary (name-bearing) type specimen in his description, both his “type” and “co-type” specimens are syntypes of equal nomenclatural status. In order to fix the name *prosternalis* to a single, name-bearing type, we here designate the male syntype labelled as “type” (Figure 13A), which is well prepared and agrees well with Lea’s description, as the lectotype of *Tranes prosternalis* and the “co-type” specimen as a paralectotype.

Specimens of *M. prosternalis* from north of Cairns differ slightly from those from south of Cairns by having the anterior margin of the rhombic sclerite of the endophallus broadly truncate and the apex narrowly rounded (Figure 9A–C, see blue arrows, Figure 10A), whereas in specimens from south of Cairns the anterior margin is narrowly rounded and the apex is broadly rounded to subtruncate (Figure 9D–F, see blue arrows, Figure 10B). However, the endophallic sclerite of some specimens is intermediate between these conditions, and in the absence of other significant morphological differences in both external and genital characters we interpret all populations as representing a single, somewhat variable species. More comprehensive study of specimens from all populations and the addition of genomic data should be able to refine the species delimitation.

#### 3.3.2. ***Miltotranes subopacus***(Lea, 1929)

(Figure 1C,D, Figure 2C,D, Figure 3D, Figure 4B,E, Figure 5B,E, Figure 6B,E, Figure 7B, Figure 8B,E,H, Figure 9G–L, Figure 10C, Figure 11B,E, Figure 13B and Figure 14)

*Tranes subopacus* Lea, 1929: 538 [24]—Wilson, 1993: 14 [1]; Schenkling & Marshall, 1936: 1 [25]; Zimmerman, 1994: 696 [14]; Oberprieler, 1995a: 306 [15]; Pullen et al., 2014: 289 [20].

*Miltotranes subopacus* (Lea)—Zimmerman, 1994: 696 [14]; Oberprieler, 1995a: 307, 329 [15]; 1995b: 338 [16]; Jones, 2002: 52 [8]; Wilson, 2001: 21 [2]; 2002b: 440 [4]; 2004: 57 [5]; Pullen et al., 2014: 289 [20]; Hsiao & Oberprieler, 2020a: 369 [6]; Toon et al., 2020: 1044 [23].

Type locality: Byfield, Queensland, Australia.

##### Material Examined

Types. Holotype (Figure 13B), ♀: “*subopacus* / Lea, TYPE / Byfield // Specimen / figured / ECZ // *Tranes* 17015 / *subopacus* Lea / Queensland / TYPE // Severely / denuded, Most / setae abraded / Det. 92 E.C. Zimmerman // HOLOTYPE / *Tranes subopacus* / Lea, 1929 / Hsiao & Oberprieler 2022” (SAMA).

Other Material. Queensland: Rockhampton, (no date) G. Wilson leg. (9 ♂, 10 ♀, 1 larva, ANIC); same data, *Tranes subopacus* c.w.t., E.C. Zimmerman (1 ♀, ANIC); Stockyard Pt., Byfield, 22°47′ S 150°47′ E, I–III.1993, A. Walford leg. (1 ♀, UQIC).

##### Diagnosis

Adults of this species can be readily distinguished from its congeners by the following characters (states of *M. prosternalis* and *M. wilsoni* in parentheses): completely dark brown body colour (Figure 1C,D) (orange to dark red, with black stripe on elytra; Figure 1A,B,E,F); longer rostrum, ca. 8.3× as long as wide in male, 9.5× in female, with antennae inserted in middle of rostrum in male (Figure 2C), slightly behind middle in female (Figure 2D) (ca. 6.0–6.3× as long as wide in male, 6.5–7.1× in female, slightly before middle in male (Figure 2A,E), in middle in female (Figure 2B,F)); intermesocoxal process with anterior margin truncate, disc even, without protuberant anterior angles (Figure 5E) (anterior margin shallowly concave, anterior angles protuberant in male (Figure 5D,F)); protibiae in male more elongate and slender, length ca. 7.4× width (Figure 6B) (shorter and thicker, length ca. 6.0× width in *M. prosternalis*; Figure 6A); abdominal ventrite 5 distinctly depressed posteriorly in female (Figure 7B) (even, without depression; Figure 7A,C); sternite VIII without median sclerite on apical margin in male (Figure 8B) (with a small rounded sclerite on apical margin medially in male (Figure 8A,C)), slender, linear, slightly curved basolaterally in female (Figure 8E) (strongly angled basolaterally in female (Figure 8D,F)); penis gradually narrowing apicad in apical third, apex rounded (Figure 9G,H,J,K) (more abruptly narrowing apicad in apical fifth, apex obtusely pointed; Figure 9A,B,D,E,M,N,P,Q); endophallus: copulatory sclerite rounded, distal patch of dentate sclerites symmetrical (Figure 10C) (copulatory sclerite rhombic, crescent-shaped distal patch of dentate sclerites asymmetrical; Figure 10A,B,D).

##### Redescription

Shape and size. Body broadly oval (Figure 1C,D), length 6.6–8.3 mm in both sexes, width ca. 0.4–0.5× length, moderately convex in lateral view (Figure 2C,D).

Colour and vestiture. Body and legs dark brown, without lustre (Figure 1C,D); body and legs covered with coarse, sublanceolate and subsquamiform, yellowish setae, clustered in parts to somewhat obscure derm, especially on pronotum, prosternal elevated process in male, scutellar shield, intermesocoxal process in male and elytra, setae longer on pronotum, prosternal elevated process in male, intermesocoxal process in male and elytra, setae denser at margins of prosternal elevated process in male.

Head. Rostrum: moderately long, longer in female (ca. 1.2× longer than pronotum in male, 1.6× in female), robust (ca. 8.3× as long as wide in male, 9.5× in female), downcurved, dorsoventrally flattened, slightly broadened apically in dorsal view, coarsely punctate dorsally, punctures slightly smaller in distal half, proximal half with paired dorsomedian and dorsolateral carinae, the latter lower than the former. Eyes: subcircular in outline, slightly convex but not protruding in dorsal view (Figure 1C,D). Antennae: inserted in middle of rostrum in male (Figure 2C), slightly behind middle in female (Figure 2D); scapes not reaching eye; funicles with segment 1 longest, ca. 1.7×, 2.5×, 2.5×, 2.6×, 2.3× and 2.2× longer than segments 2 to 7, respectively; clubs elongate, ca. 1.9× longer than wide, densely and finely pubescent (Figure 3D).

Thorax. Pronotum: roundly trapezoidal, apex ca. 0.5× narrower than base (Figure 4B); anterior margin subtruncate, slightly emarginate medially, posterior margin protruding medially, forming obtuse median lobe, lateral margins mostly rounded but distinctly converging anteriad; disc weakly and evenly convex; surface distinctly punctorugulose, with median longitudinal ridge. Prosternum: with a large, pentagonal, elevated, erect-setose patch in male, with a small process in the middle of anterior edge (Figure 4E); prosternellum elongate, widened posteriorly (Figure 5B). Mesoventrite: intermesocoxal process trapezoidal, with anterior margin truncate, disc even, without protuberant anterior angles (Figure 5E). Scutellar shield: roundly subpentagonal. Elytra: ca. 2.2–2.4× longer than pronotum, jointly ca. 0.7× as broad as long, broader than base of pronotum; humeri broadly rounded, slightly protruding; surface uneven, deeply and coarsely punctate in rows forming distinct striae, interstriae convex. Legs: femora with small ventral subapical tooth; tibiae with premucro smaller than uncus; protibiae stronger and more curved, with well developed tibial brush in male (Figure 6B); meso- and metatibiae with distal setal combs long but restricted to apical margin (Figure 6E); tarsi with claws free, divergent. Abdomen: ventrite 5 even, without depression in male, distinctly depressed in female (Figure 7B).

Terminalia. Male: sternite VIII subtrapezoidal, sclerotised except medially, apical margin rounded to truncate, without median sclerite, basal margin strongly sclerotised (Figure 8B); spiculum gastrale widely concave apically, base lightly sclerotised (Figure 8H); tegmen with complete ring, manubrium slightly shorter than parameroid lobes (Figure 9G–L); penis thick (ca. 2.1× longer than wide), subparallel-sided, gradually narrowing apicad in apical third, apex rounded, with lateral pair of dark sclerotised patches in penis wall (Figure 9G,H,J,K); endophallus with a complex of sclerites inside penis composed of basal membranous sleeve of denticles, median rounded rhombic sclerite with basal fenestra and distal field of sclerotised denticles (Figure 10C). Female: sternite VIII slender, linear, slightly curved laterally (Figure 8E); gonocoxites thick, short, apically bluntly rounded (Figure 11B); gonostyli short, conical, bluntly rounded and setose apically; bursa copulatrix without bands of spicules; spermatheca thick, right-angled, gland small, narrower than spermatheca, elongate, narrowing apicad (Figure 11E).

##### Distribution

**Figure 14:**
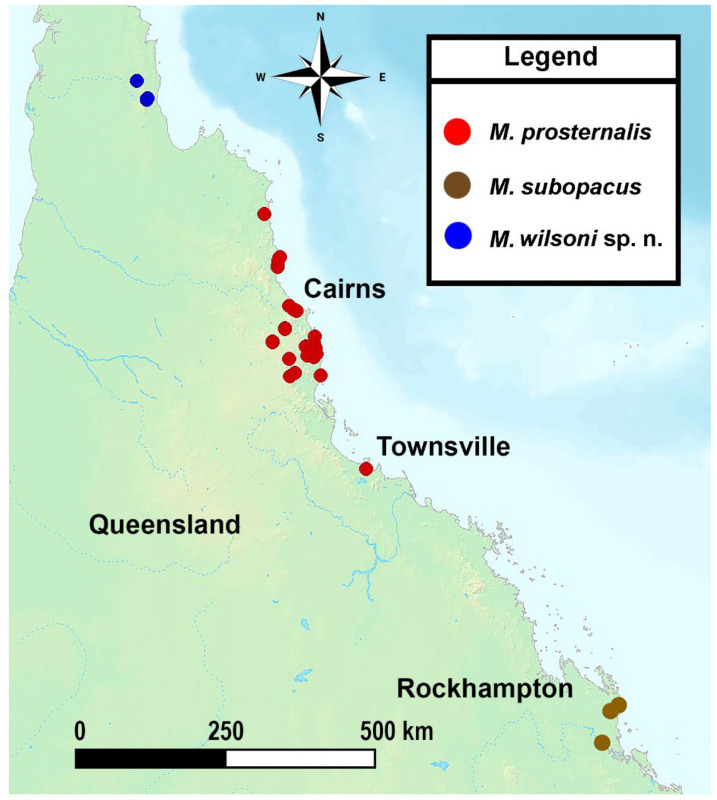
Geographical distribution of *Miltotranes* in central to northern Queensland in Australia.

*Miltotranes subopacus* only occurs in the Byfield area of central-eastern Queensland (Figure 14).

##### Natural History

*Miltotranes subopacus* is a host-specific pollinator of *Bowenia spectabilis*. Its life history is seemingly similar to that of *M. prosternalis*, according to the literature [1,2,4,5].

##### Remarks

Lea [24] described *Tranes subopacus* based on a single specimen from Byfield in Central Queensland, explicitly stating that “…the type appears to be a female” and “…the type is probably a female”. There is also only such a single specimen in his collection (in SAMA) (Figure 13B), indicating that this specimen is the holotype.

#### 3.3.3. ***Miltotranes wilsoni*** Hsiao & Oberprieler, sp. n.

(Figure 1E,F, Figure 2E,F, Figure 3E, Figure 4C,F, Figure 5C,F, Figure 6C,F, Figure 7C, Figure 8C,F,I, Figure 9M–R, Figure 10D, Figure 11C,F and Figure 14)

Zoobank Registration: http://zoobank.org/urn:lsid:zoobank.org:act:30CDC116-E969-45AF-8683-0F18E4AF0A7F

Type locality: Leo Creek, Queensland, Australia.

##### Material Examined

Types. Holotype, ♂: “21.June.1995 / AU: F.N. QLD: McILWraith Range: / Leo Creek / P. Forster leg. / Foliage of *Bowenia spectabilis* // HOLOTYPE / *Miltotranes wilsoni* / Hsiao & Oberprieler 2022” (ANIC). Paratypes (all labelled “PARATYPE / *Miltotranes wilsoni* / Hsiao & Oberprieler 2022”: Queensland: 2 ♀: “21. VIII. / 13°44′30′’S. 143°22′15′’E / Leo Creek Mine area, McIlwraith Ran- / ge, QLD. AU” (ANIC); 1 ♀: “13.45S 143.22E QLD / 8 km WbyN of Bald Hill / McIlwraith Range / 27 June–12 July 1989 / T.A. Weir 500 m / mango tree site // *Miltotranes* / N.Sp. / teneral? ♀ / Det. **‘**94 E.C. Zimmerman” (ANIC); 5 ♂, 2 ♀: same data as holotype (ANIC); 9 ♂, 12 ♀: “21.June.1995 / AU: QLD: 13°44′ S. 143°22′ E, Leo / Creek mine area, Timber Reserve 14, / McILWraith Range / P. I. Forster. leg. (Voucher: P.I. Forster / PIF16831) / Grazing on young expanding fronds of / *Bowenia spectabilis*” (ANIC).

##### Diagnosis

This species is externally very similar to *M. prosternalis* but differs from it as detailed in the diagnosis of *M. prosternalis* above.

##### Redescription

Shape and size. Body broadly oval (Figure 1E,F), length 5.6–7.0 mm in both sexes (6.8 mm in holotype), width ca. 0.5× length, moderately convex in lateral view (Figure 2E,F).

Colour and vestiture. Head dark red, rostrum black, antennae reddish brown, thorax orange to dark red, elytra orange to dark red with black anterior margin and anterior half of elytral interval 1 and an irregular black macula medially, abdomen dark red, coxae, trochanters, femora and tibiae black, tarsi reddish brown, semilustrous (Figure 1E,F); body and legs covered with coarse, sublanceolate and subsquamiform, yellowish setae, clustered on prosternal elevated process in male and intermesocoxal process in male, somewhat obscuring derm, setae longer on pronotum, prosternal elevated process in male, intermesocoxal process in male and elytra, setae denser in margins of prosternal elevated process in male.

Head. Rostrum: moderately long, longer in female (ca. 1.4× longer than pronotum in male, 1.6× in female), robust (ca. 6.3× as long as wide in male, 7.1× in female), downcurved, dorsoventrally flattened, slightly broadened apically in dorsal view, coarsely punctate dorsally, punctures slightly smaller in distal half, proximal half with paired dorsomedian and dorsolateral carinae, the latter lower than the former. Eyes: subcircular in outline, slightly convex but not protruding in dorsal view (Figure 1E,F). Antennae: inserted slightly before middle of rostrum in male (Figure 2E), in middle in female (Figure 2F); scapes not reaching eye; funicles with segment 1 longest, ca. 1.6×, 2.4×, 2.6×, 2.6×, 2.2× and 2.1× longer than segments 2 to 7, respectively; clubs elongate, ca. 1.8× longer than wide, densely and finely pubescent (Figure 3E).

Thorax. Pronotum: roundly trapezoidal, apex ca. 0.6× narrower than base (Figure 4C); anterior margin subtruncate, slightly emarginate medially, posterior margin protruding medially, forming obtuse median lobe, lateral margins mostly rounded but distinctly converging anteriad; disc weakly and evenly convex; surface distinctly punctorugulose, with median longitudinal ridge. Prosternum: with a large, subrhombic (anteriorly rounded), elevated, erect-setose patch in male, with a small process anteriorly (Figure 4F); prosternellum elongate, posteriorly widened and truncate (Figure 5C). Mesoventrite: intermesocoxal process trapezoidal, with anterior margin shallowly emarginate and anterior angles protuberant in male (Figure 5F). Scutellar shield: roundly subpentagonal. Elytra: ca. 2.2–2.4× longer than pronotum, jointly ca. 0.7× as broad as long, broader than base of pronotum; humeri broadly rounded, slightly protruding; surface uneven, deeply and coarsely punctate in rows, forming distinct striae, interstriae convex. Legs: femora with small ventral subapical tooth; tibiae with premucro smaller than uncus; protibiae stronger and more curved, with well developed tibial brush in male (Figure 6C); meso- and metatibiae with distal setal comb long but restricted to apical margin (Figure 6F); tarsi with claws free, divergent. Abdomen: ventrite 5 even, without depression in both male and female (Figure 7C).

Terminalia. Male: sternite VIII subtrapezoidal, sclerotised, apical margin rounded to truncate, apical margin with a small rounded sclerite medially, basal margin strongly sclerotised (Figure 8C); spiculum gastrale widely concave apically, base lightly sclerotised (Figure 8I); tegmen with complete ring, manubrium slightly shorter than parameroid lobes (Figure 9M–R); penis thick (ca. 1.6–1.7× longer than wide), subparallel-sided, distinctly narrowing apicad in apical fifth, apex obtusely pointed (Figure 9M,N,P,Q); endophallus with a complex of sclerites inside penis composed of basal membranous sleeve of denticles, median elongate rhombic sclerite and distal crescent-shaped patch of dense denticles (Figure 10D). Female: sternite VIII abruptly angled laterally (Figure 8F); gonocoxites thick, short, apically bluntly rounded (Figure 11C); gonostyli short, conical, bluntly rounded and setose apically; bursa copulatrix without bands of spicules; spermatheca thick, right-angled, gland small, narrower than spermatheca, elongate, narrowing apicad (Figure 11F).

##### Derivation of Name

The species is named for Gary Whittaker Wilson, botanist at the Australian Tropical Herbarium at James Cook University, who undertook a significant major study of the pollination and systematics of *Bowenia*.

##### Distribution

*Miltotranes wilsoni* is known from only two localities in the Mcllwraith Range in northern Queensland (Figure 14) and appears to be restricted to a small area east of the Mungkan Kandju National Park, where its *Bowenia* hostplant occurs.

##### Natural History

No specific information is available about the life history of the species, but it is presumably very similar to those of the other two *Miltotranes* species, especially that of *M. prosternalis*. Most of the specimens were collected from young expanding fronds or foliage at the end of June, which is the cooler season, rather than the summer coning period. This suggests that the species may overwinter in the adult stage, which agrees with a similar presumption made for *M. prosternalis* by Hsiao & Oberprieler [6].

##### Remarks

The taxonomic status of *M. wilsoni* as a species distinct from *M. prosternalis* was first noted by Elwood Zimmerman in 1994 (see Material Examined above), and Oberprieler later provisionally supported it (pers. comm. 2000 to G. Wilson [5]). These observations are confirmed in the present study, based on numerous differences from *M. prosternalis* in the characters of both external and genital structures. These differences are in line with species differentiations in other genera of the *Tranes* group [12,26].

## 4. Discussion

### 4.1. Systematic Placement of Miltotranes and Evolution of Cycad Pollination in the Tranes Group

*Miltotranes* belongs to the systematically enigmatic *Tranes* group of genera [14], which has been recently placed in the tribe Orthorhinini [21], based on the phylogenomic study of Shin et al. [27], or in its own tribe, Tranini, based on morphological characteristics [22]. *Miltotranes* is one of two genera of the *Tranes* group pollinating their cycad hosts, and its species are the sole obligate pollinators of *Bowenia*. Despite the as yet unresolved phylogenetic relationships in the *Tranes* group, *Miltotranes* has a close affinity to *Tranes* based on morphological characters including colour pattern (dark brown to reddish brown; Figure 1), position of the scrobes (parallel to rostrum in lateral view; Figure 2) and sexually dimorphic prosternum (prominently protuberant in the males; Figure 4) and protibiae (with a large brush in the males; Figure 6), which suggests that the obligate cycad pollination systems of *Tranes* and *Miltotranes* may represent a single evolutionary event in the *Tranes* group. The cycad host genera of *Tranes*, *Macrozamia* and *Lepidozamia*, are not closely related to *Bowenia* [28,29,30], and *Macrozamia* and *Lepidozamia* are also not each other’s closest relatives (the latter being more closely related to the African genus *Encephalartos*), so that evolutionary host shifts are indicated to have occurred both in *Tranes* and in the *Tranes* group. Given the distant and evidently much older phylogenetic origin of *Bowenia* [28,29,30], Oberprieler [11] earlier already suggested that the association of *Miltotranes* with *Bowenia* represents a secondary colonisation of this genus by the *Tranes* group. A phylogenetic analysis using genomic data is currently in preparation to resolve the systematic position and phylogenetic relationships of *Miltotranes* and the evolution of its cycad pollination.

### 4.2. Implications of Miltotranes Systematics on Bowenia Taxonomy

The genus *Bowenia* is endemic to tropical Queensland and currently contains two described extant species, *B. spectabilis*, occurring mainly in the Wet Tropics bioregion of northeast Queensland (from Cape Melville in the north to Cardwell in the south), and *B. serrulata*, restricted to the Byfield area of the Central Queensland Coast bioregion (north of Rockhampton). Whereas the species limits of *B. serrulata* have not been contentious, the taxonomic status of a number of populations of *B. spectabilis* has been uncertain, in particular an isolated northern one in the McIlwraith Range of the Cape York Peninsula but also another seemingly isolated one in the Starcke National Park north of Cooktown and two on the Atherton Tablelands, one at Kuranda and the other at Tinaroo. All have been treated as “putative *B. spectabilis*” by Wilson [5], and the Tinaroo population has even been regarded as a distinct species [8,31]. As *Miltotranes* weevils are known from all these populations and act as the pollinators of the plants, their species identities have implications for the taxonomic status of these cycad populations.

The localised northern *Bowenia* population in the McIlwraith Range is geographically separated from the *B. spectabilis* populations further south by the Normanby Gap, also known as the Laura Gap or Laura Basin, a drier, alluvial-lowland river catchment area that separates the Iron Range and McIlwraith Range rainforest area of the Cape York Peninsula from the Wet Tropics [32]. This geographical disjunction is believed to be of Late Miocene age [5], but its exact age and progression over time requires further investigation. The McIlwraith *Bowenia* population is usually treated as belonging to *B. spectabilis* [7,33], but Wilson [5], having had insufficient samples of it available for his morphological study, only provisionally regarded it as belonging to this species. Kokubugata et al. [34,35], using a cytotaxonomic approach to address the taxonomic issues of *Bowenia*, were also unable to include this population in their karyotype analyses, and no rigid genetic analysis has been conducted of it to date. Its taxonomic status therefore remains somewhat uncertain.

Wilson [5] also regarded the *Bowenia* population in the Starcke National Park as an isolated one, separated from populations further south by the Black Mountain Divide or Corridor (located just north of Cairns [32]). There are, however, other *Bowenia* populations north of this corridor (e.g., at Cape Tribulation, Cooktown and Cape Melville), and it does not appear to be a barrier of significance for *Bowenia*.

The status of the *Bowenia* populations at Kuranda and Tinaroo has been uncertain due to their seemingly intermediate morphological characteristics between *B. serrulata* (pinnules with serrate margin; caudex large and branched) and *B. spectabilis* (pinnules with entire margin; caudex small and sparsely branched). The Tinaroo population has been treated as a distinct, undescribed species [8,31], whereas Norstog & Nicholls [36] regarded it only as an infraspecific variety of *B. spectabilis*. Hill & Osborne [7] found that serrate pinnules occur in all populations of *B. spectabilis*, and Wilson [5] concluded from his comprehensive morphological study that pinnule and caudex structure are phenotypically plastic characters determined by ecological factors (especially temperature) and that the Kuranda and Tinaroo populations are likely only ecotypes rather than subspecies of *B. spectabilis*. This conclusion is supported by the karyotype analyses of Kokubugata et al. [34,35], which revealed that these two populations have the same number of median-centromeric chromosomes as *B. spectabilis* and are thus cytotaxonomically closer to this species than to *B. serrulata*.

From our study of *Miltotranes* weevils from these three populations and also from the Endeavour River at Cooktown (ca. 60 km south of the Starcke population) we conclude that the specimens from the Kuranda, Tinaroo and Cooktown populations of *Bowenia* are conspecific and represent *M. prosternalis* (the Endeavour River being its type locality) and also that specimens from all populations of *B. spectabilis* further south (from Cape Tribulation to Townsville) represent the same species. Only the specimens from the isolated northern McIlwraith population represent a different species, here described as *M. wilsoni* and being distinguishable from *M. prosternalis* on several morphological features, including of the genitalia.

The conspecificity of *Miltotranes* weevils from the Kuranda, Tinaroo and Cooktown populations (and thus likely also the Starcke population) of *Bowenia* indicates that these plant populations are also conspecific and all represent *B. spectabilis*. In contrast, the recognition of a different species of *Miltotranes* occurring in the McIlwraith population of *Bowenia* (*M. wilsoni*) supports the notion that this population may not represent *B. spectabilis* [5] but a different species as well. It further suggests that this cycad population and its weevil pollinator may have become isolated together from their congeneric populations further south by the development of the Normanby Gap and that their concomitant differentiation may represent a case of co-speciation mediated by vicariance. Likewise, the (evidently older) evolutionary divergence of the species pair of *M. prosternalis* and *M. wilsoni* from *M. subopacus* in the south may be concomitant with that of *B. spectabilis* from *B. serrulata* and could also be due to vicariance (the development of the dry-savanna Burdekin and Saint Lawrence Gaps [32]). However, analysis of the phylogeny of the *Tranes* group and circumspect research into the timing of the diversification events in both the weevils and the plants is required to explore these scenarios.

## 5. Conclusions

This first systematic revision of the weevil genus *Miltotranes*, whose species are the sole known pollinators of the small endemic Australian cycad genus *Bowenia*, results in the identification and delimitation of three species, the previously described *M. prosternalis* and *M. subopacus* and a newly recognised and described species, *M. wilsoni*. Several morphological characters of *Miltotranes* shared with *Tranes* indicate that these two genera are closely related (evidently sister taxa) and, given the widespread association of *Tranes* with the cycad genus *Macrozamia* but also with the two species of *Lepidozamia* (which is more closely related to the African *Encephalartos*), it appears that the association of *Miltotranes* with *Bowenia* may represent an evolutionary host shift in the *Tranes* group from *Macrozamia* to *Bowenia*.

The ranges of the three *Miltotranes* species coincide well with those of their cycad hosts, *M. subopacus* only occurring on the southern species *B. serrulata*, *M. prosternalis* on the northern *B. spectabilis* and *M. wilsoni* on the northern-most and isolated *Bowenia* population in the McIlwraith Range of the Cape York Peninsula, which is thus indicated to represent a distinct, third species of *Bowenia*. Similarly, the conspecificity of the *Miltotranes* weevils occurring in the Cooktown/Starcke, Kuranda and Tinaroo populations with *M. prosternalis* suggest that these populations also represent *B. spectabilis*, confirming earlier botanical conclusions that the latter two only represent morphologically slightly different ecotypes [5,7]. Furthermore, the taxonomic and geographical congruence between the three species of *Miltotranes* and their *Bowenia* hosts suggests that their evolutionary differentiations may also be concomitant.

The recognition of a new species of *Miltotranes* occurring only in the McIlwraith population of *Bowenia* indicates that the conservation status of this population warrants closer attention. Both *B. spectabilis* and *B. serrulata* are included in the IUCN Red List as species of Least Concern and CITES Appendix II, and the localised McIlwraith taxon of *Bowenia* and its specific weevil pollinator, *M. wilsoni*, may be in need of similar protection and recognition in international inventories of threatened species.

This study lastly highlights the relevance of systematic study of tightly plant-associated insects for the taxonomy of their hosts. As the only known pollinators of *Bowenia* cycads, *Miltotranes* weevils are evidently instrumental in maintaining the reproductive integrity of their hosts, and their species identities are therefore also highly relevant for the species identities of their hosts. The case of *Bowenia* cycads and their *Miltotranes* pollinators emphasises the need for more comprehensive and congruent taxonomic and phylogenetic studies of the plants and their associated weevils.

## Data Availability

The data underlying this article are available in Zenodo at https://zenodo.org/ and can be accessed with http://doi.org/10.5281/zenodo.6451541, accessed on 16 April 2022.
